# Is it necessary for young patients with recurrent implantation failure to undergo preimplantation genetic testing for aneuploidy?

**DOI:** 10.3389/fendo.2023.1020055

**Published:** 2023-02-01

**Authors:** Yulin Du, Yichun Guan, Na Li, Congxing Shi, Yongjie Zhang, Bingnan Ren, Jing Liu, Hua Lou

**Affiliations:** ^1^ Reproductive Center, The Third Affiliated Hospital of Zhengzhou University, Zhengzhou, Henan, China; ^2^ School of Public Health, Sun Yat-sen University, Guangzhou, China

**Keywords:** recurrent implantation failure, preimplantation genetic testing for aneuploidy, blastocyst, frozen embryo transfer, pregnancy outcomes

## Abstract

**Objective:**

To determine whether preimplantation genetic testing for aneuploidy (PGT-A) can improve the pregnancy outcomes of patients aged under 38 years who have a history of recurrent implantation failure(RIF).

**Design:**

Retrospective cohort study.

**Methods:**

We retrospectively studied the pregnancy outcomes of RIF patients aged under 38 years from January 2017 to December 2021.178 patients were divided into two groups according to whether they underwent PGT-A: the PGT-A group(n=59)and the control group(n=119).In the PGT-A group, we compared the euploidy rate of the different quality and developmental rate blastocysts. In both groups,the patients were the first frozen-thaw single blastocysts transfer after the diagnosis of RIF. Among the pregnancy outcomes, the clinical pregnancy rate was assessed as the primary outcome. The spontaneous abortion rate and ongoing pregnancy rate were the secondry outcomes. The generalized estimation equation was used to adjust for the blastocysts derived from the same patients. Multivariate logistic analysis models were used to compare the pregnancy outcomes between the two groups.

**Results:**

In the PGT-A group, 293 blastocysts obtained from59 patients underwent PGT-A. The proportions of euploidy, aneuploidy and mosaic blastocysts were 56.31%, 25.60% and 18.09%, respectively. A comparison of the euploidy rates of different quality blastocysts showed that the rate of good-quality blastocysts was significantly higher than that of poor-quality blastocysts (67.66% vs 46.88%; odds ratio [OR], 2.203; 95%confidence interval[CI], 0.943–3.612; P=0.002). However, no significant difference was observed in the different developmental rates blastocysts. Compared with Day 5 blastocysts, the euploidy rates of Day 6 and Day 7 blastocysts were not significantly different(61.54%vs51.91%; OR,0.945; 95%CI, 0.445–2.010; P=0.884; and 61.54%vs47.37%; OR, 1.106; 95%CI, 0.774–1.578; P=0.581, respectively).As for the pregnancy outcomes, the clinical pregnancy rate was significantly increase after the use of PGT-A compared with the control group(71.19%vs56.30%; OR, 0.538; 95%CI, 0.262–1.104; P=0.039). However, the spontaneous abortion rates and ongoing pregnancy rates were not significantly different between the control and PGT-A groups (21.43% vs 19.40%; aOR,0.727; 95%CI,0.271–1.945; P=0.525; and55.93% vs 45.38%; aOR, 0.649; 95%CI, 0.329–1.283; P = 0.214,respectively).

**Conclusion:**

PGT-A improved the clinical pregnancy rate after blastocyst transfer in RIF patients aged under 38 years.

## Introduction

While the success rate of *in vitro* fertilization–embryo transfer (IVF-ET) has improved significantly in the past 40 years, many couples still experience the frustration of multiple failed embryo transfer attempts for reasons that are not yet clear. This phenomenon, often described as recurrent implantation failure (RIF), has become a major challenge in the field of assisted reproduction. The cause of RIF remains a black box for clinicians to explore. While this clinical phenomenon is commonly encountered and a vast literature is available on this subject, there is still no universally accepted definition of RIF. Several studies in the literature have described RIF as “the failure to achieve a clinical pregnancy after transfer of at least 4 good-quality embryos in a minimum of three fresh or frozen cycles in a woman under the age of 40 years.” (1).

Embryo implantation is a process in which embryos with developmental potential are implanted into the receptive endometrium. Several factors may contribute to inefficient implantation, leading to RIF, one of which is the rate of chromosomal aneuploidy. Traditional morphological assessment commonly used to screen embryos cannot determine the ploidy status of the embryos, particularly because the association between the morphology and ploidy status of embryos is not perfect (2). It is worth noting that the aneuploidy rate is strongly related to age. A recent study demonstrated a remarkable difference in the aneuploidy rates among women of different age groups, ranging from 30% to 50% in women younger than 35 years of age to as high as 80% in women aged 42 years (3).

Preimplantation genetic testing for aneuploidy (PGT-A) is the definitive tool for embryo selection on the basis of ploidy status (4). It is mainly recommended for patients with advanced maternal age, RIF, recurrent pregnancy loss or severe male infertility (5). In theory, euploid blastocyst transfer has many advantages. A recent series of clinical trials reported a significant improvement in implantation and delivery rates and a reduction in the spontaneous abortion rates and time to pregnancy in different categories of patients who underwent PGT-A compared with those who underwent conventional IVF (6). Euploidy embryo transfer is thought to optimize outcomes in couples with infertility. However, there is an study indicated that PGT-A may be detrimental for women aged under 38 years undergoing their first IVF cycle (7). In previous studies, evidence supporting the efficacy of this approach in managing RIF is also insufficient, there were different views on the clinical value of PGT-A for patients with RIF. Fodina and colleagues study the RIF patients whose median age were 35 years, the PGT-A group showed statistically significant higher chance in achieving biochemical and clinical pregnancy (8). In Cozzolina’s study, moderate RIF patients were received at least three embryos transferred in different single embryo transfers (SET) without achieving implantation. Severe RIF patients underwent at least five embryo transferred. They considered that PGT-A may be beneficial for patients with moderate RIF but not for severe cases among patients aged 18-45 years (9). And among the RIF patients under 40 years, a statistically signifcant increase in the implantation rate per transfer as well as the live birth rate per embryo transfer was observed (10). And the Kato’s study showed that among the patients with RIF aged 35-42 years there were no signifcant diferences in the pregnancy outcomes (11). Jing Tong, et al. explored the value of PGT-A in the clinical outcomes for the RIF patients with advanced age. They suggested that the component of aneuploidy embryos rate was significantly higher in the advanced age group while there were no statistically differences in clinical outcomes (12). And a recent multi-center, prospective pilot study of PGT-A versus expectant management of cases of RIF found that PGT-A did not improve the live birth rates per patient or decrease the rate of the miscarriage per clinical pregnancy (13). As far as the current studies were concerned, there are few studies on clinical value of PGT-A for young RIF patients, aged <38years.

Therefore, the purpose of this study was to compare pregnancy outcomes after PGT-A, on the basis of a combination of morphological criteria, with those on the basis of morphological criteria only, with the aim to provide further clinical guidance on embryo selection for patients aged under 38 years who have a diagnosis of RIF.

## Materials and methods

### Study setting and patients

**Figure 1:**
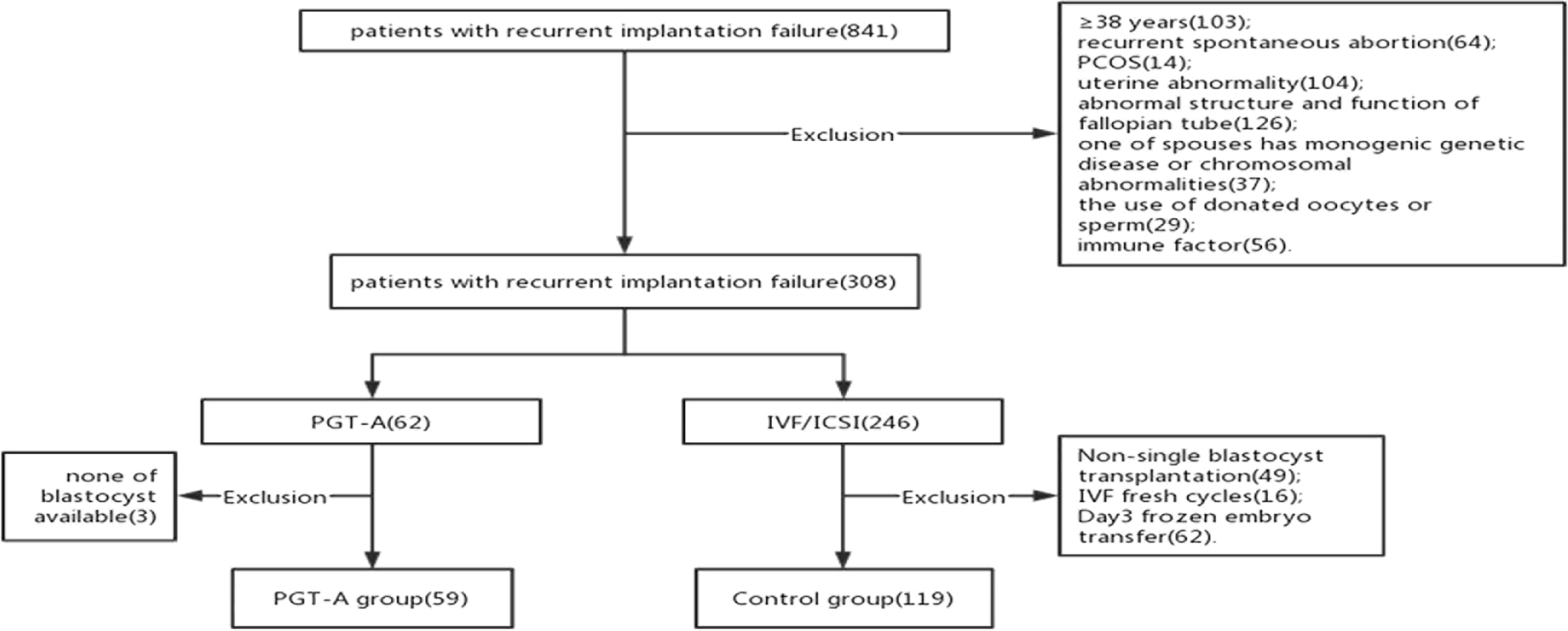
Flow chart of study process.

Our cross sectional research included patients who were diagnosed RIF at the Reproductive Center of the Third Affiliated Hospital of Zhengzhou University between January 2017 to December 2021. We include the patients RIF were and <38 years. We adopted the following criteria of RIF that more than or equal to 3 fresh or frozen transfer cycles or accumulate more than or equal to 4 high quality cleavage embryos or 2 good quality blastocysts (14). All the patients were the first IVF and frozen-thaw single blastocysts transfer cycle after the diagnosis of RIF. And we analysis the pregnancy outcomes of the first FET cycle. And the patients were excluded if they had a known uterine abnormality, immune dysfunction, coagulation abnormalities, endocrine diseases, one of the spouses has monogenic genetic diseases or chromosomal abnormalities, donated oocytes or sperm cycles, azoospermia, the endometrial thickness on transfer day <7mm, the female has a diagnose of diminished ovarian reserve(DOR)and recurrent spontaneous abortion. Finally, a total of 178 patients were included in this retrospective study (**Figure 1**). The patients were divided into two groups according to with or without PGT-A: the PGT-A group(n=59) and the control group(n=119).

### Treatment protocols

#### Controlled ovarian hyperstimulation and oocyte retrieval

All participants were treated with a flexible GnRH antagonist protocol. Ovarian stimulation was initiated on cycle day 3, with use of daily injections of 150-300IU of gonadotropin, and when dominant follicles grew to 12-14mm, subcutaneously injecting 0.25mg of GnRH antoganist(Cetrotide, Merck-Serono, Switzerland)was used for suppressing LH surge. And 0.2 mg of GnRH-A was injected subcutaneously at 23: 00 on the trigger day. Oocyte retrieval was performed 33-36h later, cumulus–oocyte complexes were collected, and oocytes were denuded (15).

### IVF/ICSI procedures

In the PGT-A group, ICSI was performed to ensure the high fertilization rates and to avoid any contamination caused by the attachment of residual sperm-derived DNA to the zona pellucida at biopsy. In the control group, IVF or ICSI fertilization method was selected according to the semen quality on the day of oocytes retrieval and a combination of previous medical history. Embryos were cultured at 37°C in 5% O_2_, 6% CO_2_ G-1 Plus medium after insemination. Fertilization was assessed at 17–20 h later, which was considered normal when two distinct pronuclei were visible. and the embryos were cultured in an incubator for 5–7 days under 5% CO_2_/5% O_2_/90% N_2_ at 37°C (15). A morphologic score was assigned to blastocyst-stage embryos according to the Gardner criteria (16).Blastocysts with the morphological score of AA,AB,BA,BB were considered as good quality and blastocysts with grading lower than BB were considered as poor quality (17).

### Blastocysts biopsy

Blastocysts above the 4BC level were laser punched and crinkled, and trophectoderm cells away from the inner cell mass were selected for biopsy. The blastocysts were fixed with holding pins closer to the endocytic mass, keeping the endocytic mass away from the biopsy. A laser is used to punch a hole on the opposite side of the fixed blastocyst, approximately 30-40µm in diameter, and the biopsy needle is used to aspirate 3-6 trophoblast ectodermal cells and drag them out of the zona pellucida notch, and a laser is used to sever the cells from the tight junctions and with blunt cutting to separate them from the blastocyst as a whole. The biopsied TE cells were washed three times in hosphate-bufered saline (PBS), transferred to a PCR tube containing 2.5 μL of PBS, and cryopreserved at −80°C until analysis (13). After biopsy, the blastocysts were transferred to G-2 Plus culture medium, and then they were kept by vitrification.

### Gene amplification and sequencing by next-generation sequencing (NGS)

Whole genome amplification was performed by SurePlex method, 7.5μllysate Mix was added to the sample tubes for cell lysis, 5µl of pre-amplification Mix was added to each lysis tube to obtain pre-amplification products, 60µl of amplification Mix was added to the pre-amplification product tube, amplification was performed on a PCR instrument, and 2µl of amplification products were taken to detect the concentration by Qubit 3.0.

The amplification products were purified, and the data were analyzed after sequencing with the Illumina Hiseq 2500, an ultra-high-throughput sequencing system. Copy number variation (CNV) analysis was performed to compare with the reference baseline. Blastocysts are judged to be aneuploid without deviation. The result is a scatter plot.Decipher, Clinvar, and UCSC databases are used to search and interpret CNVs. The Decipher database records the chromosomal location, pure and heterozygous status, pathogenicity, and phenotype of each CNV case; the Clinvar database records the genes where CNVs are located, pathogenicity, and other information; the UCSC database can check whether a specific CNV is located in functional region of the gene. NGS sequencing reports the classification of results: whole ploidy, aneuploidy and chimeras (mosaic).Percentages of aneuploid DNA in a single biopsy are used to differentiate between euploid (<20%), mosaic(21-80%) and aneuploid(>80%) embryos.

### Frozen-thawed embryo transfer

All the blastocysts in the both groups underwent vitrification freezing, warming and frozen embryo transfer. Three FET protocols are used at our center - natural cycles, hormone replacement therapy (HRT) cycles and down-regulation cycle. For natural cycles, women with normal ovulation received a trigger shot of human chronic gonadotropin or no medication during FET cycles. And for the patients with abnormal ovulation, the HRT cycle and down-regulation cycle were underwent. They were treated with estrogen and progesterone with or without prior down-regulation with gonadotropin-releasing hormone agonist.

All the patients in the both groups underwent frozen-thaw single blastocysts transfer. In the both groups, the blastocysts with highest morphological scores were selected to transfer. And in the PGT-A group, the euploidy blastocysts were transferred. Progesterone were used to luteal phase support.

### Outcome measures

Primary outcome was clinical pregnancy rate. Clinical pregnancy rate is defined as the proportion of FET cycles resulting in intrauterine gestational sacs visualized on transvaginal ultrasound. Sub-analysis outcomes included spontaneous abortion rate and ongoing pregnancy rate. Spontaneous abortion rate is defined as a pregnancy ending in the spontaneous loss of the embryo or fetus before 12 weeks of gestation. Ongoing pregnancy rate was defined as the proportion of a viable pregnancy with a fetal heartbeat after 12 weeks of gestation. Demographic data were also collected for FET cycle.

### Statistical methods

The generalized estimation equation(GEE)was used to control the effects of repeated measurements of the multiple blastocysts of the same patients in the results of euploidy rate among different morphological scores blastocysts, such as good quality blastocysts and poor quality blastocysts and different developmental blastocysts.

Comparative analyses were planned to assess differences in FET outcomes between patients with RIF undergoing FET cycles with and without PGT-A. Categorical variables were compared with the chi-square and Fisher exact tests. Continuous variables were expressed as mean ± SD and were tested for normality. Student t test was used for parametric data. The multivariate logistic analysis was used to investigate the effect of PGT-A on the pregnancy outcomes after eliminating confounding factors such as maternal age, infertility factors and the peak endometrial thickness on clinical pregnancy rate, spontaneous abortion rate and ongoing pregnancy rate. Odds ratios (ORs) with 95% confidence intervals (CIs) were calculated to control for confounding. *P*<0.05 was considered to be statistically significant. All analyses were performed with use of SPSS 26.0 statistical software(IBM, United States).

## Results

**Figure 2:**
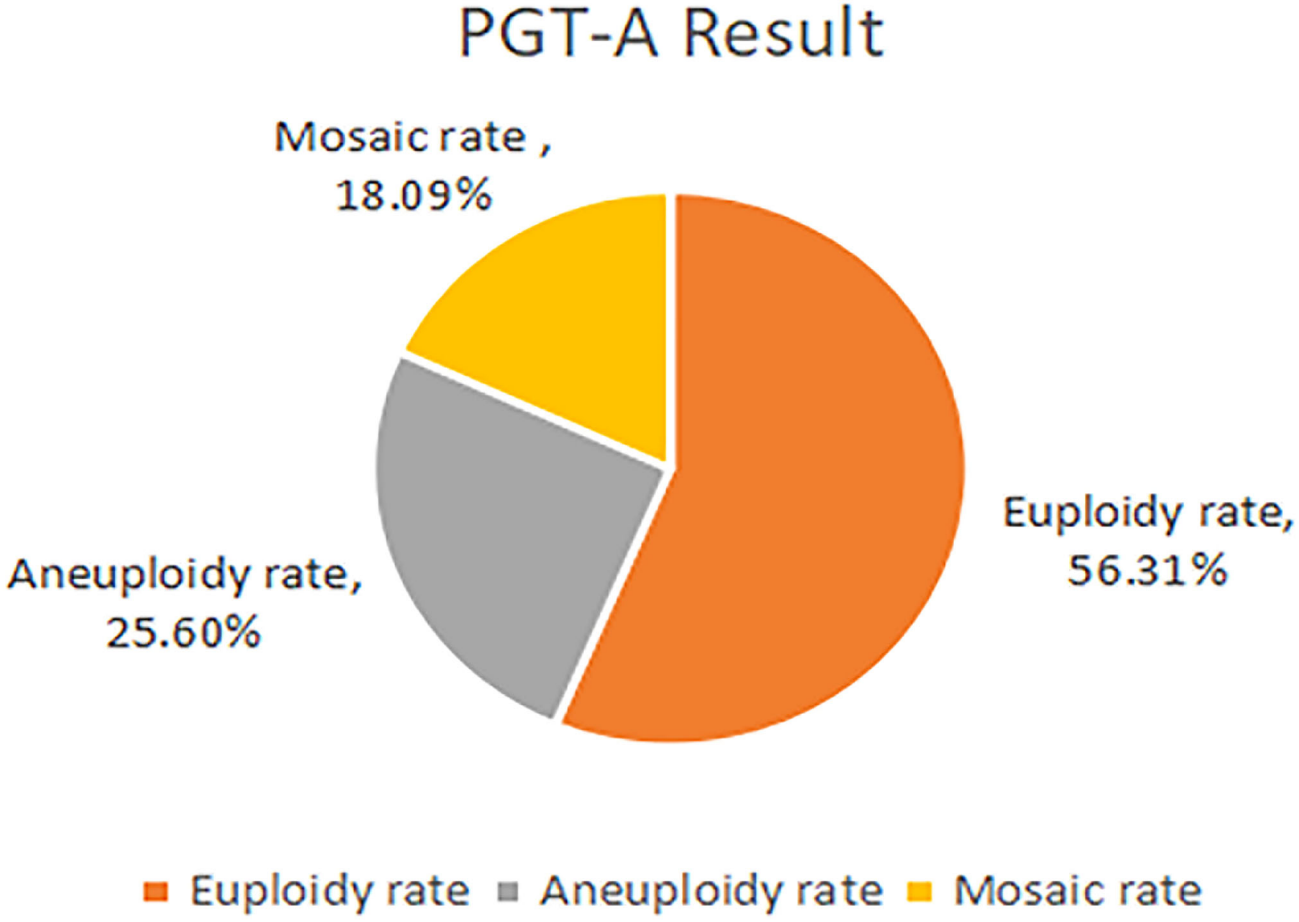
The result of PGT-A.

178 patients with a history of RIF were enrolled in the study according to the inclusion and exclusion criteria. These patients were divided into two groups according to whether they underwent PGT-A: the PGT-A group (n = 59) and the control group (n = 119)(**Figure 1**). 302 blastocysts were obtained from the 59 patients. Among them 9 blastocysts failed to amplify. The successful expansion rate was 97.02%.(293/302). Of these, 165 (56.31%) blastocysts were found to be euploid, while 75 (25.60%) and 53 (18.09%) were aneuploid and mosaic, respectively (**Figure 2**). The blastocyst biopsy results showed that good-quality blastocysts had a higher euploidy rate than poor-quality blastocysts (67.66% vs 46.88%; aOR, 2.203; 95% CI, 0.943–3.612; *P* = 0.002). These odds remained significant after adjusting for all confounders. Furthermore, compared with Day 5 blastocysts, the euploidy rates of Day 6 and Day 7 blastocysts were not significantly different (61.54% vs 51.91%; OR, 0.945; 95% CI, 0.445–0.967; *P* = 0.884; and 61.54% vs 47.37%; OR, 1.106; 95% CI, 0.516–829; *P* = 0.713, respectively) (**Table 1**).

**Table 1:** Blastocyst biopsy results of PGT-A group.

Variable	Value	Euploidy rate	P Value	OR (95%CI)
Blastocysts quality	Good quality	67.66(90/133)		
	Poor quality	46.88(75/160)	0.002*	2.203(0.943-3.612)
Developmental rate	Day5	61.54(88/143)		
	Day6	51.91(68/131)	0.884	0.619(0.445-0.967)
	Day7	47.37(9/19)	0.581	0.713(0.516-0.829)

Adjusted for *P < 0.05 was considered to be statistically significant.

Baseline characteristics are detailed in **Table 2**. In terms of maternal age, patients in the PGT-A group were older than those in the control group (32.64 ± 2.03 years vs 31.45 ± 3.50 years; *P* = 0.018). Furthermore, the infertility types were significantly different between the two groups. The patients in the PGT-A group were more likely to the secondary infertility (67.80% vs 44.54%; *P* = 0.004). Compared with the control group, the endometrial thickness on transfer day was significantly thinner in the PGT-A group(8.75 ± 1.38 mm vs 9.85 ± 1.73 mm; *P*<0.001). The quantity of good-quality blastocysts and the developmental rate of blastocysts were not significantly different in the both groups (*P* = 0.337 and *P* = 0.258, respectively).

**Table 2:** Baseline demographics of PGT-A group and control group.

	PGT-A group (n=59)	Control group (n=119)	P
Maternal age(year)	32.64 ± 2.30	31.45 ± 3.50	0.018*
Maternal body-mass index(kg/m2)	22.96 ± 2.53	23.10 ± 2.83	0.754
Paternal age(year)	33.68 ± 4.67	32.27 ± 5.09	0.076
Duration of infertility(year)	2.59 ± 1.12	2.50 ± 0.53	0.472
Previous times of implantation failures(time)	2.81 ± 2.46	3.27 ± 1.73	0.144
Ifertility factors(%)			0.341
female factor	52.54(31/59)	54.62(65/119)	
male factor	13.56(8/59)	18.49(22/119)	
Female-male factor	15.25(9/59)	10.92(13/119)	
unexplained factor	18.64(11/59)	15.97(19/119)	
Infertility types(%)			0.004*
primary infertility	32.20(19/59)	55.46(66/119)	
secondary infertility	67.80(40/59)	44.54(53/119)	
Anti-Mullerian hormone(ng/ml)	3.56 ± 2.24	3.50 ± 2.33	0.876
Follicle-stimulating hormone(IU/liter)	6.77 ± 2.10	6.28 ± 2.37	0.183
Antral folicle count in both ovaries	17.39 ± 6.55	17.24 ± 5.60	0.877
Endometrial preparation(%)			0.070
Natural cycle	55.93(33/59)	39.50(47/119)	
Hormone replacement therapy cycle	32.20(19/59)	36.97(44/119)	
Down-regulation cycle	11.86(7/59)	23.53(28/119)	
Endometrial thickness on transfer day(mm)	8.75 ± 1.38	9.85 ± 1.73	<0.001*
Blastocysts of development days(%)			0.258
D5	62.71(37/59)	49.58(59/119)	
D6	33.90(20/59)	46.22(55/119)	
D7	3.39(2/59)	4.20(5/119)	
The quantity of good quality blastocyst	62.71(37/59)	54.62(65/119)	0.337

*P < 0.05 was considered to be statistically significant.

As for the pregnancy outcomes, a multivariate logistic regression was performed after adjusting for confounding factors, including maternal age, type of infertility and endometrial thickness on transfer day. The clinical pregnancy rate was significantly higher in the PGT-A group than in the control group (71.19% vs 56.30%; aOR, 2.538; 95% CI, 1.398–6.746; *P* = 0.039) (**Table 3**). However, no significant difference was found in the spontaneous abortion rate between the two groups (21.43% vs 19.40%; aOR, 0.727; 95% CI, 0.271–1.945; *P* = 0.525). Similarly, no difference was observed in the ongoing pregnancy rate in the two groups (55.93% vs 45.38%; aOR, 0.649; 95% CI, 0.329–1.283; *P* = 0.214) (**Table 3**).

**Table 3:** Clinical outcomes between PGT-A group andontrol group.

	PGT-A group	Control group	OR	P
Clinical pregnancy rate	71.19 (42/59)	56.30 (67/119)	2.538(1.398-6.746)	0.039*
Spontaneous abortion rate	21.43 (9/42)	19.40 (13/67)	0.727(0.271-1.945)	0.525
Ongoing pregnancy rate	55.93 (33/59)	45.38 (54/119)	0.649(0.329-1.283)	0.214

*P < 0.05 was considered to be statistically significant. Odds ratios (ORs) with 95% confidence intervals (CIs) were calculated and adjusted for confounder factors such as maternal age, infertility types and the peak endometrial thickness for each patient.

## Discussion

This study aimed to explored whether the PGT-A can improving the pregnancy outcomes of the patients aged under 38 years with a diagnosis of RIF. Our data show that the good-quality blastocysts had higher euploidy rate than that in the poor-quality blastocysts. While the findings also indicate that the euploidy rate was not correlated with the blastocyst developmental rate. As for the pregnancy outcomes, our data present that the clinical pregnancy rate was significantly higher in the PGT-A group than that in the control group. Compared with the control group which were selected by morphological score, there were no significant difference in the spontaneous abortion rate and the ongoing pregnancy rate between the two groups.

In this study, the good-quality blastocysts had a higher euploidy rate than that in the poor-quality blastocysts. As the previous studies showed, the good-quality blastocysts showed a higher percentage of euploidy (2, 18, 19) and the poor-quality blastocysts had reduced the rate of euploidy (20). The euploidy blatocysts had intact chromosome and the chromosomes specific mechanisms leading to euploidy or aneuploidy. So the euploidy blastocysts are more likely to develop with higher morphological scores. Our findings also showed that there was no significant difference in the euploidy rate of the different developmental rate. The blastocyst developmental rate may be influenced by not only the difference in aneuploidy rates but also other factors, such as the metabolic or epigenetic health of the blastocysts.

American Society for Reproductive Medicine(ASRM)point out that PGT-A is used to patients who had a high risk of aneuploidy blastocysts. Advanced maternal age patients have more opportunity to produce aneuploidy blastocysts and PGT-A can select euploidy blastocysts to transfer, improving their pregnancy outcomes (4). According to the present study, the clinical value of PGT-A is still controversial. There are still different opinions on whether PGT-A should be used in advanced age patients. It has been suggested that routine PGT-A for the selection of euploid embryos may improve delivery rates in these older women (21). As we all know, decline in oocyte quality is associated with aneuploidy, at the same time the quality of blastocysts in elderly patients is low and the capacity of DNA repair is inadequate, and the DNA repair function is impaired, resulting in reduced development potential. Thus it can be seen that PGT-A is an effective way to assist reproduction for advanced maternal age patients. In patients with RIF, the effect of PGT-A is also disputable. However, some studies have shown that PGT-A has not improved the clinical outcome of elderly patients. And the Kato’s study showed that among the patients with RIF aged 35-42 years, because of the poor endometrial environment, the implantation rate of aneuploid blastocysts transplantation may not be improved (11). The embryo damage caused by biopsy and the false negative rate of the results are also important reasons for the pregnancy outcome not being improved (12). Compared with the moderate RIF patients, severe cases may have other pathogeny, such as endometrial receptivity (9).The PGT-A may represent a valuable supplement to the current infertility patients management, while whether PGT-A is underwent or not requires clinicians to choose individualized treatment plan according to the actual situation, such as the patient’s age, ovarian reserve and severity of illness. And there were few studies discussing the PGT-A in patients with RIF, <38years.

In this study of which the patients were <38 years, RIF, another important observation is that the clinical pregnancy rate was significantly higher in the PGT-A group than that in the control group. However, the use of PGT-A did not decrease the spontaneous abortion rate. The ongoing pregnancy rate has no significant difference between the two groups, either.

The benefit of PGT-A when selecting the blastocysts transfer has been demonstrated in many studies. As the previous studies showed, for the patients with advanced maternal age, transferring a euploid embryo could improved the pregnancy outocmes (6, 7, 22, 23). The clinical pregnancy rate and live birth rate can be improved using PGT-A. The PGT-A selected the euploidy blastocysts to transfer, improving the adverse pregnancy outcomes caused by aneuploidy rate. It is common knowledge that the patients ≥38 years whose blastocysts quality were poorer have lower euploidy rate than that in younger patients (18).

Despite the positive outcomes in many studies, others have not shown PGT-A to improve pregnancy outcomes. Chen’s article in New England magazine pointed out that conventional IVF resulted in the pregnancy outcome that was noninferior to the rate with PGT-A in the patients with good prognosis (12). In the patients<38 years, many studies found no statistically significant difference in the PGT-A group versus controls (22, 24). In our study, the spontaneous abortion rate and ongoing pregnancy rate had no significant difference in the both groups. As our knowledge, the etiology of RIF has not yet been revealed completely. Maybe for the younger patients there are other reasons why the spontaneous abortion rate has not decreased. And due to technical limitations of the process, the blastocysts biopsy may cause the blastocysts damage. The security of PGT-A is also the focus of current research. Another thing to consider is that the rate of false positive results of euploidy blastocysts has been reported (16). This maybe another reason why the spontaneous abortion rate did not decrease with the use of PGT-A.

In this study, the PGT-A group had a higher clinical pregnancy rate than that in the control group. It seems solve the clinical pregnancy rate of RIF patients. But some patients are still no achieving clinical pregnancy. There may be other undetected causes. Moreover, the aneuploid blastocysts is not necessarily an embryo with implantation potential. We should explore other methods and indicators, and conduct clinical and basic experiments, so that we can provide other treatments for patients.

This study has several advantages. Our research object is patients<38 years with RIF. This study has strict inclusion and exclusion criteria, and we excluded RIF patients with abnormal coagulation or immune function. And we only examined frozen-thaw single blastocysts transfer cycles to avoid the confounding effects of any differences in fresh IVF versus FET outcomes.

This study also has several limitations. First, the bias caused by the limitations of retrospective cohort study itself. Second, the sample size was small while it is also representative. Third, the study did not evaluate the cost-effectiveness of PGT-A for informing clinical decisions. And our future studies would track the live birth rates of patients over a longer period. In addition, we did not analyze the failure times and severity.

## Conclusion

PGT-A improved the clinical pregnancy rate in RIF patients aged under 38 years compared with IVF-FET without PGT-A, but had no effect on the spontaneous abortion rate and ongoing pregnancy rate.

## Data availability statement

The original contributions presented in the study are publicly available. This data can be found here: https://data.mendeley.com/datasets/wc35zm64ym/1.

## Ethics statement

The studies involving human participants were reviewed and approved by Ethics Committee of the Third Affiliated Hospital of Zhengzhou University. Written informed consent for participation was not required for this study in accordance with the national legislation and the institutional requirements.

## Author contributions

HL and YG proposed the design ideas, YD, CS, and BR acquired and analyzed the data, NL, YZ, and JL prepared all tables and figures, YD wrote the manuscript, HL revised the manuscript. All authors contributed to the article and approved the submitted version.
